# Bisphenol-A Neurotoxic Effects on Basal Forebrain Cholinergic Neurons In Vitro and In Vivo

**DOI:** 10.3390/biology12060782

**Published:** 2023-05-28

**Authors:** Andrea Flores, Paula Moyano, Emma Sola, José Manuel García, Jimena García, María Teresa Frejo, Lucia Guerra-Menéndez, Elena Labajo, Inés Lobo, Luisa Abascal, Javier del Pino

**Affiliations:** 1 Departamento de Farmacología y Toxicología, Facultad de Veterinaria, Universidad Complutense de Madrid, 28040 Madrid, Spainluisaab94@hotmail.com (L.A.);; 2 Departamento de Fisiología, Facultad de Medicina, Universidad San Pablo CEU, 28003 Madrid, Spain; 3 Departamento de Medicina Legal, Psiquiatría y Patología, Facultad de Medicina, Universidad Complutense de Madrid, 28040 Madrid, Spain

**Keywords:** bisphenol-A, basal forebrain, cholinergic neurons, synaptic plasticity, histone deacetylase 2, WNT/β-Catenin pathway, neurodegeneration

## Abstract

**Simple Summary:**

Environmental pollutants have been suggested to be among the possible causes that play a role in the generation of Alzheimer’s disease (AD) and other neurodegenerative diseases. Included in these environmental pollutants, we find the highly used plasticizer bisphenol-A, which produces neurodegeneration and cognitive disorders similar to those induced in AD. However, the mechanisms through which this and other environmental pollutants produce these effects are unknown. In AD, as well as in other neurodegenerative diseases that produce cognitive disorders, a selective cholinergic neuronal loss is induced in the brain region of the basal forebrain, which in turn leads to the denervation of the hippocampus and cortex, producing neurodegeneration in these regions and, eventually, cognitive disorders. Our results show the alteration of some mechanisms that could mediate bisphenol-A disruption of synaptic plasticity and neurodegeneration induction in this specific type of basal forebrain neurons. These results may assist to elucidate the processes that mediate the cognition alterations produced by bisphenol-A and other environmental pollutants, which it shares mechanisms with, and could lead to the development of preventive and therapeutic tools to avoid and treat these effects in the population.

**Abstract:**

The widely used plasticizer bisphenol-A (BPA) is well-known for producing neurodegeneration and cognitive disorders, following acute and long-term exposure. Although some of the BPA actions involved in these effects have been unraveled, they are still incompletely known. Basal forebrain cholinergic neurons (BFCN) regulate memory and learning processes and their selective loss, as observed in Alzheimer’s disease and other neurodegenerative diseases, leads to cognitive decline. In order to study the BPA neurotoxic effects on BFCN and the mechanisms through which they are induced, 60-day old Wistar rats were used, and a neuroblastoma cholinergic cell line from the basal forebrain (SN56) was used as a basal forebrain cholinergic neuron model. Acute treatment of rats with BPA (40 µg/kg) induced a more pronounced basal forebrain cholinergic neuronal loss. Exposure to BPA, following 1- or 14-days, produced postsynaptic-density-protein-95 (PSD95), synaptophysin, spinophilin, and N-methyl-D-aspartate-receptor-subunit-1 (NMDAR1) synaptic proteins downregulation, an increase in glutamate content through an increase in glutaminase activity, a downregulation in the vesicular-glutamate-transporter-2 (VGLUT2) and in the WNT/β-Catenin pathway, and cell death in SN56 cells. These toxic effects observed in SN56 cells were mediated by overexpression of histone-deacetylase-2 (HDAC2). These results may help to explain the synaptic plasticity, cognitive dysfunction, and neurodegeneration induced by the plasticizer BPA, which could contribute to their prevention.

## 1. Introduction

Bisphenol-A (BPA), a plasticizer used wide world, produces cognition disorders in animals, following acute and continuous exposure [1,2], and is also associated with this effect in humans, as described in epidemiological studies [3]. Although some of the BPA actions involved in these effects have been unraveled, several are still incompletely known.

BPA has been shown to increase the expression of histone deacetylase 2 (HDAC2), which partially impairs cholinergic transmission and induces basal forebrain (BF) cholinergic SN56 neuronal death by disrupting the nerve-growth-factor (NGF)/p75-neurotrophin-receptor (P75^NTR^)/tropomyosin-receptor-kinase-A (TrkA) pathway and through acetylcholinesterase-S overexpression, following 1- or 14-days of treatment [4]. In addition, BPA exposure disrupts the insulin pathway, produces oxidative stress, and promotes the accumulation of beta-amyloid (Aβ) and tau peptides, in part, through HDAC2 and protein tyrosine phosphatase 1 (PTP1B), an insulin pathway regulator and overexpression, contributing to the apoptosis observed in the cholinergic cell line SN56 [5].

Cognitive functions are maintained by neuronal circuits, which originate in the basal forebrain cholinergic neurons (BFCN) and end in the hippocampus and frontal cortex [6,7]. When BFCN neurodegeneration occurs, there is a subsequent denervation of these circuits that leads to hippocampal and cortical neuronal loss, resulting in cognitive disabilities [7,8,9]. Therefore, BPA-induced cholinergic neurotransmission disruption or the neurodegeneration produced in SN56 BFCN, if produced in vivo may alter hippocampal and cortical circuits, leading to the cognition dysfunction described. However, the studies described above have only been developed in an in vitro model from BFCN, but no study has ever been developed to show the effects of BPA on BFCN in vivo. Therefore, it is necessary to determine whether BPA induces selective or more pronounced neuronal cell death in BFCN in vivo, and when corroborated, further in vitro studies in SN56 cells will be required to particularly elucidate all neurotoxic mechanisms specifically involved in BFCN.

BPA is also known for disrupting synaptic plasticity, which contributes to cognitive dysfunction, through HDAC2 overexpression [10]. However, the mechanisms through which HDAC2 produces this effect are unknown. BPA alters the protein expression of postsynaptic density protein 95 (PSD95), spinophilin (SPN), synaptophysin (SYP), and N-methyl-D-aspartate receptor subunit 1 (NMDAR1) synaptic proteins, known for regulating synaptic plasticity and cognitive function [1,2,11]. BPA was also shown to disrupt glutamatergic transmission, by affecting either glutamate uptake or its synthesis, which is related to synaptic plasticity disruption, neurodegeneration, and cognitive dysfunction [12,13,14,15]. Finally, BPA was described to downregulate the WNT/β-Catenin pathway, which is related to cell death, synaptic plasticity disruption, and cognitive dysfunction [16,17,18]. HDAC2 overexpression, induced by different compounds other than BPA and pathological situations, was shown to disrupt all the mechanisms commented on above [19,20,21].

Therefore, we hypothesize that BPA induces selective or more pronounced BFCN loss, and could produce glutamatergic transmission dysfunction, synaptic proteins expression downregulation, and WNT/β-Catenin pathway disruption through HDAC2 overexpression in this specific type of neurons that may lead to cell death. To test this hypothesis, firstly, 60 days-old male Wistar rats were exposed to a single dose of BPA (40 µg/kg) and sacrificed after 48 h; and secondly, SN56 cells, a model of BFCN, either silenced or not against *Hdac2* and/or *Ptp1b* were treated with BPA (0.001 µM to 1 µM), either alone or in combination with MK-801 (NMDAR1 antagonist; 20 µM) and/or recombinant β-Catenin (rβ-Catenin, 15 µM), for 1- or 14-days.

## 2. Materials and Methods

### 2.1. Animal Treatment Protocol

The animal research was performed according to guidelines established by European Union (2010/63/EU) and the Spanish RD 53/2013 that regulates the research animals’ welfare. Male Wistar rats (60-days and approximately 200 g of weight) were acquired from Charles River (Barcelona, Spain) to develop the present research. Rats were housed individually allowing them to freely access food and water and the room conditions were 22 ± 2 °C temperature and a photoperiod of 12 h darkness/12 h light. Animals were randomly distributed into two groups (6 animals/group) which were treated subcutaneously once with either BPA (40 µg/kg) or physiological saline solution, as a control group, and were sacrificed 48 h after treatment, since it has been reported histological alterations in the brain are visible approximately two days after the exposure to toxic compounds [22]. In order to avoid bias, a blinded procedure was developed in the studies performed.

We selected the dose of 40 µg/kg since it was previously reported that this single dose, when administered subcutaneously to Wistar adult male rats, induces synaptic plasticity dysfunction, decreases PSD95 levels, and produces cognitive dysfunction [1]. Furthermore, this dose is relevant for human toxicity since it is below the BPA reference dose settled by the United States Environmental Protection Agency (50 μg/kg/day) [1].

### 2.2. Basal Forebrain Preparation for Histopathological Studies

A craniotomy was developed, and cerebrums were extracted, intact, for histopathological studies (n = 6 for control and treatment groups). Formalin buffer (10%) was employed for three days to fix the brains, which were sectioned and processed following guidelines of the National Toxicology Program [23], and a standard rodent brain atlas [24]. The brain sections were embedded in paraffin, cut in 5 µm thickness slices for eosin and hematoxylin (H-E) staining (Dako CoverStainer, Agilent, Spain) [25], using a rotatory microtome, and evaluated under a microscope (Nikon E200, Madrid, Spain).

Following the published guidance, we singled out two BF sections using the “Bregma coordinate” system, which is presented in millimeters (mm), with positive (rostral) or negative (caudal) values depending on their location in relation to the zero Bregma plane. The basal forebrain nuclei studied in our research were the medial septal nucleus (MSN), the diagonal band of Broca (DBB), and the nucleus basalis of Meynert (NBM). Bregma +0.84 mm section was selected to study the MSN and the DBB (vertical and horizontal limbs) and Bregma −1.44 mm was selected to analyze the nucleus basalis of Meynert. H-E-stained sections were used to delineate anatomical landmarks for each BF nucleus, in consonance with published studies [24,26,27].

### 2.3. Immunohistochemistry Studies

To examine the brain tissue using immunohistochemistry, a fully automated platform from Roche (Madrid, Spain) called Bench-Mark ULTRA was employed. Firstly, 4 µm coronal slices of 4 µm thickness, as previously recommended for performing immunohistochemistry [28], were deparaffinized, and then heat-induced antigen retrieval was performed in citrate buffer at pH 6 at 91 °C for 56 min (for ChAT antibody) or 24 min (for NeuN antibody). The brain sections were then incubated with the primary antibodies NeuN (Clone A60, MAB377, Sigma, Madrid, Spain) and ChAT (clone JA67-11, NBP2-66779, NovusBio, Centennial, CO, USA) with a dilution 1/100 for 30 min at 37 °C. After rinsing, the sections were incubated with biotinylated secondary antibodies for 30 min, followed by streptavidin-biotin peroxidase complex. Signal amplification was performed using the Ventana amplifier kit Ultra Wash (Roche, Madrid, Spain), and then the sections were counterstained with hematoxylin and bluing reagent. The UltraView Universal DAB detection kit from Roche (Madrid, Spain) was employed to detect chromogenic changes.

### 2.4. Histopathological Study

The histological changes induced by BPA were analyzed in the indicated neuronal regions from BF mentioned earlier. Specifically, necrotic neurons defined by contracted and dark-pigmented pyknotic nuclei [29], were analyzed on every type of neuron, as well as on cholinergic neurons, using the marker ChAT. The previously delineated BF nuclei were subjected to quantitative analysis to assess the extent of neuronal necrosis. Additionally, viable neurons were studied using the immunohistochemical marker NeuN [30]. The NeuN marked neuron quantification was performed in all delineated BF nuclei commented above.

### 2.5. Culture Procedure

The BFCN SN56 cell line [31], kindly gifted by Professor Laura Calzà (CIRI-SDV and Fabit, University of Bologna), was used as an archetype of BF cholinergic neurons to determine the specific harmful effects produced on cholinergic neurons after BPA (239658; Sigma, Madrid, Spain) treatment and the processes that mediated their induction. The culturing procedure was performed according to Moyano et al. [4]. Every forty-eight hours, the medium was discarded and a freshly made one was added [32]. We differentiated SN56 cells, as previously described in the literature [33,34], resulting in a fold increase of acetylcholine content and ChAT activity (3 to 4 times fold) after three days of culture with the maintenance medium described above without FBS and containing 1 µM retinoic acid (R2625; Sigma, Madrid, Spain) and 1 mM dibutyryl-cAMP (D0627; Sigma, Madrid, Spain), obtaining cells which were more sensitive to cholinergic neurotoxicity [33,34] We tested that cells were mycoplasma free with the LookOut Mycoplasma kit from Sigma (MP0035; Madrid, Spain).

In order to determine phosphorylated-GSK3β (p-GSK3β; ser9), WNT3a, β-Catenin, Cyclin D1, c-Myc, PSD95, SPN, SYP, NMDAR1, PTP1B, HDAC2, glutaminase, glutamate, and vesicular glutamate transporter 2 (VGLUT2) protein content, glutaminase, and GSK3β activity, *Gsk3β, Psd95, Spn, Syp, Nmdar1, Wnt3a, Β-Catenin, Cyclin D1, C-Myc, Vglut2*, and *glutaminase* gene expression, *Hdac2* and *Ptp1b* gene knockdown effects, cell viability, and apoptosis induction, differentiated cells (passages 7–15) were seeded in 6-well plates (2 × 10^6^ or 10^6^ cells/well density for 24 h or 14 days treatment, respectively) or 96-well (4000 or 2000 cells/well for 24 h or 14 days treatment, respectively) in the above-described maintenance medium and exposed to BPA (0.001 µM to 1 µM) either for 1-one- or fourteen-days and co-treated with MK-801 (NMDAR1 antagonist; 20 µM; M107; Sigma, Madrid, Spain) and/or recombinant β-Catenin (rβ-Catenin, 15 µM; 23-027; Sigma, Madrid, Spain) or vehicle. Treatments were added once the media was changed for a fresh one, every 48 h. BPA was solved in 0.1% dimethyl sulfoxide (DMSO; C6164; Sigma, Madrid, Spain) as a vehicle. A negative control, with only the vehicle, was carried out. The data obtained from the controls showed no statistical differences between 1- or 14-days exposure, so they were combined and represented as a single white bar in the figures of each study. A minimum of 3 replicate wells/treatments were performed.

BPA is present, as reported in the literature, in human biological samples (around 0.001 µM to 1 µM) [35,36,37,38], showing that humans are widely exposed to it. BPA higher levels have been detected in handlers (higher and continuous contact) or just after contact with the compound; reaching probably the highest levels in organs with higher lipids content, such as the brain, where it accumulates [39,40]. The range of BPA (0.001 µM to 1 µM) concentrations chosen for this study is in accordance with those with which the majority of humans may be in contact with and that were tested to study the processes through which BPA induces its harmful effects on in vitro studies, and cognitive disorders in animal studies [41,42,43]. Lastly, 0.1 µM BPA concentration was chosen, since it reduces the viability of cells, disrupts both NGF/P75^NTR^/TrkA and insulin pathways, and increases Aβ and phosphorylated-tau protein levels, following unique exposure in SN56 cells [4,5], to research the suggested pathways that could mediate SN56 neurodegeneration after BPA single and continuous exposure. The indicated rβ-Catenin and MK-801 concentrations were selected as these were the lowest concentrations that activate the WNT/β-Catenin pathway or completely block NMDAR1, respectively.

### 2.6. Protein Determination

Once the treatments were applied, cells were lysed and homogenized according to Moyano et al. [4]. Total protein concentration was analyzed in the homogenized supernatant using a BCA kit (23227; Thermo-Fisher Scientific, Madrid, Spain).

Protein content quantification of p-GSK3β (Ser9), WNT3a, β-Catenin, Cyclin D1, c-Myc, PSD95, SPN, SYP, NMDAR1, PTP1B, HDAC2, and VGLUT2 proteins was determined with ELISA commercial kits (MBS9501465, MBS2881507, MBS724736, MBS9312804, MBS7725905, MBS1607968, MBS9717307, MBS7724834, MBS8807324, MBS753717, MBS2507320, and MBS1605765, respectively, MyBioSource, San Diego, CA, USA), following the procedure indicated by the manufacturers. A negative control, to avoid BPA interferences with the kit reagents, was conducted through ELISA assays in which cell homogenates were not added. Since protein quantity is proportional to the number of cells, the concentration of all target proteins indicated above was normalized with the absolute protein concentration determined with the BCA kit to prevent the bias induced in the final results when cell death is produced. Results obtained as ng/mg of protein were expressed as a percentage of the untreated control.

### 2.7. GSK-3β Activity Analysis

The GSK-3β phosphorylation activity is determined by measuring the radioactivity of γ-phosphate radioactive residues introduced in the substrate by the GSK-3β enzyme using γ-adenosine triphosphate (ATP). GSK-3β activity was analyzed using GSK-3β Activity Assay Kit (CS0990; Sigma, Madrid, Spain), according to the producer’s procedures. GSK-3β was immunoprecipitated, in the lysed cells procured according to Moyano et al. [4], using a conjugate of agarose beads and anti-GSK-3β antibody (EZview Red Protein G Affinity Gel, P6486; Sigma, Madrid, Spain). Then, the GSK-3β obtained was incubated with substrate and ^γ32^P-ATP, which introduces ^32^P residues into the substrate. The radioactivity emitted by the ^γ32P^-substrate was quantified through scintillation counting and normalized with the absolute protein concentration as indicated above. Results obtained are presented as percentages of the untreated control.

### 2.8. Gene Expression Analysis

The procedure to perform gene expression analysis was conducted according to Moyano et al. [4] and results were analyzed according to Livak and Schmittgen [44]. Validated primers (SA Biosciences) for mRNAs encoding beta-actin (*Actb,* housekeeping gene; PPM02945B), *Psd95* (PPH01848A), *Spn* (PPM34114A), *Syp* (PPM03241A), *Wnt3a* (PPM04720C), *Nmdar1* (PPM04235A), *β-catenin* (PPM03384A), *Cyclin D1* (PPM02903F), *c-Myc* (PPM02924F), *Ptp1b* (PPM05101F), *Hdac2* (PPM04361F), and *Vglut2* (PPM35412A) were used with the SA Biosciences PCR master mix (Real-Time SYBR Green, PA-012) to run qPCR in a CFX96. A qPCR in which cDNA was not included was used as a negative control.

### 2.9. Glutamate Content

Glutamate concentration was quantified in culture medium and cell lysates following BPA exposure, according to previous studies [12]. Samples for HPLC analysis were processed by perchlorate (460494, Sigma, Madrid, Spain) precipitation and o-phthalaldehyde (P137, Sigma, Madrid, Spain) derivatization, and analyzed by HPLC with electrochemical detection, using a 5 µm particle size C18-Nucleosil reversed-phase column (4.6 mm i.d. × 150 mm; Z226173, Sigma, Madrid, Spain), with a mobile phase composed of 0.1 M Na_2_HPO_4_·2H_2_O, 0.1 M citric acid (pH 3.5) and 20% (*v/v*) methanol at +0.85 V in relation to the reference Ag/AgCl_2_ electrode, as described in the literature [45,46,47,48]. Absolute protein content detected with a BCA kit was used to normalize as indicated above the glutamate concentrations and detected results are expressed as μmol/mg of protein.

### 2.10. Glutaminase Function

Glutaminase function was determined employing the commercial Glutaminase Microplate Assay Kit (MBS8243221, MyBioSource, San Diego, CA, USA), following the producer’s guidelines and as described in previous studies [12]. Absolute protein content detected with a BCA kit was used to normalize, as indicated above, the glutaminase function, which was expressed as μmol/h/mg protein and represented as a percentage compared to the untreated control.

### 2.11. Hdac2 and Ptp1b Silencing

*Hdac2* and *Ptp1b* were silenced following the protocol established by Moyano et al. [4]. Two sets of siRNA duplexes (Qiagen, Barcelona, Spain), homologous to mouse *Hdac2* and *Ptp1b* sequences for each of them were acquired from Qiagen (GS19246 and GS15182, respectively). An All Stars Negative Control siRNA (Qiagen, Barcelona, Spain) was used as a transfection control. Following forty-eight hours of transfection, *Hdac2* and *Ptp1b* silencing efficacy was analyzed by qPCR, measuring the target genes expression, which displayed a significant decrease in their expression. Treatment protocol for transfected cells includes incubation with siRNA for twenty-four hours followed by PBS washing and further incubation with treatments scheduled.

### 2.12. Analysis of Cell Viability

The SN56 cells viability was determined following BPA treatment (1- or 14-days) using the Sigma 3-(4,5-dimethyl-2-thiazolyl)-2,5-diphenyl-2H-tetrazolium-bromide (MTT) (475989, Madrid, Spain) test. MTT yellow reagent (100 µL, 0.5 mg/mL pattern solution) was used to incubate cells for 4 h at 37 °C following BPA treatment, then culture media was discarded and DMSO (250 µL) was added to wells to solubilize the precipitated purple formazan, which was read at 570 nm using a Fluoroskan Ascent FL Microplate Fluorometer and Luminometer (Thermo Fisher Scientific, Madrid, Spain). DMSO treatment was employed as a control. Results obtained were expressed as a percentage of the untreated control.

### 2.13. Caspase Activation Determination

Caspase activation, evaluated with Caspase-Glo 3/7 luminescence assay kit (G8090, Promega, Madrid, Spain), was used to determine the presence of apoptotic cells. The assays were performed following the manufacturer’s guidelines. Briefly, at the end of exposure, cells were rinsed with PBS, scraped, and gathered in lysis buffer under dark conditions, in a microfuge tube; being an aliquot poured into a white-walled 96-well plate with the same amount of reagent and incubated in the dark for one hour at 20 °C. Samples were read in a Perkin Elmer LS50B plate-reading luminometer (Madrid, Spain) to measure their luminescence. Results obtained were expressed as a percentage of the untreated control.

### 2.14. Statistical Analysis

For in vivo studies, data are presented as mean ± standard deviation (SD). For in vitro studies, data are presented as means ± standard error of the mean (SEM) and are representative of the (leastwise) three experiments performed for each research study in triplicate (n = 9). Student’s *t*-test (single treatment comparisons), one-way (treatment-response comparisons), and two-way (transfection/treatment comparisons) ANOVA analyses followed by Tukey post-hoc test were developed to determine statistically significant differences between treatments (*p* ≤ 0.05), employing GraphPad 5.0 Software Inc. (San Diego, CA, USA).

## 3. Results

### 3.1. BPA Acute Exposure Effects on Basal Forebrain Neurons

**Figure 1:**
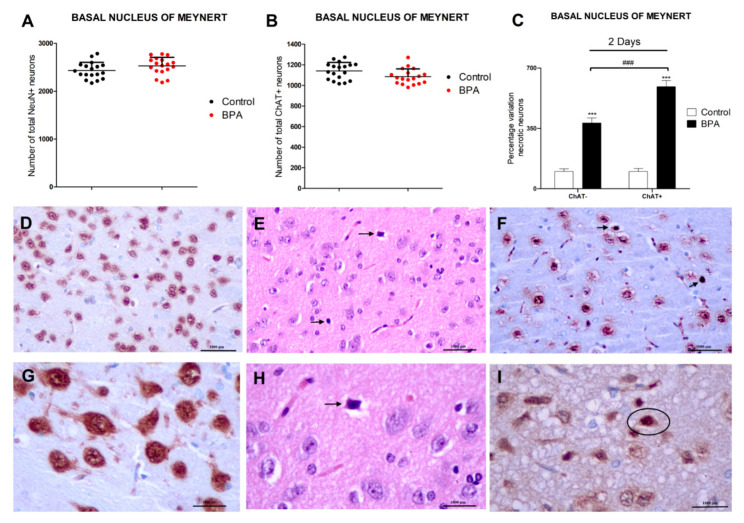
BPA effects in Wistar rats’ nucleus basalis of Meynert’s NeuN+, ChAT+, and necrotic (ChAT−/ChAT+) neurons after acute (40 µg/kg) exposure (**A**–**C**). Data represent the mean (n = 6 animals for control and treatment groups) ± SD of the number of total NeuN neurons (**A**), total ChAT+ neurons (**B**), and the percentage variation of ChAT−/ChAT+ necrotic neurons (**C**) compared to control. Three sections were used per nuclei. At least 3 replicates of each type of neuron counting were performed. Two way-ANOVA (treatment vs. type of neuron comparison) or Student’s *t*-test (control vs. treatment comparison). *** *p* < 0.001 compared to control (**C**); ^###^ *p* < 0.001 compared to ChAT− necrotic neurons (**C**). (**D**–**I**) show Wistar rat’s nucleus basalis of Meynert’s photomicrographs. NeuN immunohistochemical stain highlights NeuN+ mature neurons in (**D**,**G**) (20× and 40× magnification, respectively, scale bar = 1000 μm) and ChAT immunohistochemical stain highlights ChAT+ cholinergic neurons in (**F**,**I**) (20× and 40× magnification, respectively, scale bar = 1000 μm). (**E**,**F**,**H**,**I**) show BPA acute exposure effects, illustrating ChAT+ necrotic neurons (arrows or circle) in (**F**,**I**) (20× and 40× magnification, respectively, scale bar = 1000 μm), and necrotic neurons (arrows) in (**E**,**H**) (H-E, scale bar = 1000 μm, 20× and 40× magnification, respectively) adjacent to more normal neurons. (**E**,**H**) depict acute eosinophilic neuronal necrosis, with noticeable eosinophilia of the cytoplasm and basophilia of the nucleus.

BPA effects on basal forebrain neurons were evaluated by counting ChAT+ and NeuN+ neurons and necrotic (ChAT+ and ChAT−) neurons in the three outlined BF nuclei indicated above (Figure 1). The same results were observed in the 3 different nuclei studied (Figure 1 and Supplementary Figure S1) and there were no significant differences in the amount of ChAT+ and NeuN+ neurons, as well as in the percentages of necrotic (ChAT− and ChAT+) neurons between the 3 BF nuclei after BPA exposure (Figure 1 and Supplementary Figure S1). 

Exposure to a single dose of BPA (40 µg/kg) resulted in a significant percentage increase of necrotic (ChAT− and ChAT+) neurons in the basal forebrain. The percentage increase of necrotic ChAT+ neurons was higher than that of necrotic ChAT− neurons. The amount of NeuN+ and ChAT+ neurons was not affected by BPA treatment after a single dose exposure (Figure 1).

### 3.2. Analysis of Glutamatergic Neurotransmission (Glutamate Content, Glutaminase Activity, and VGLUT2 Protein Content and Gene Expression)

**Figure 2:**
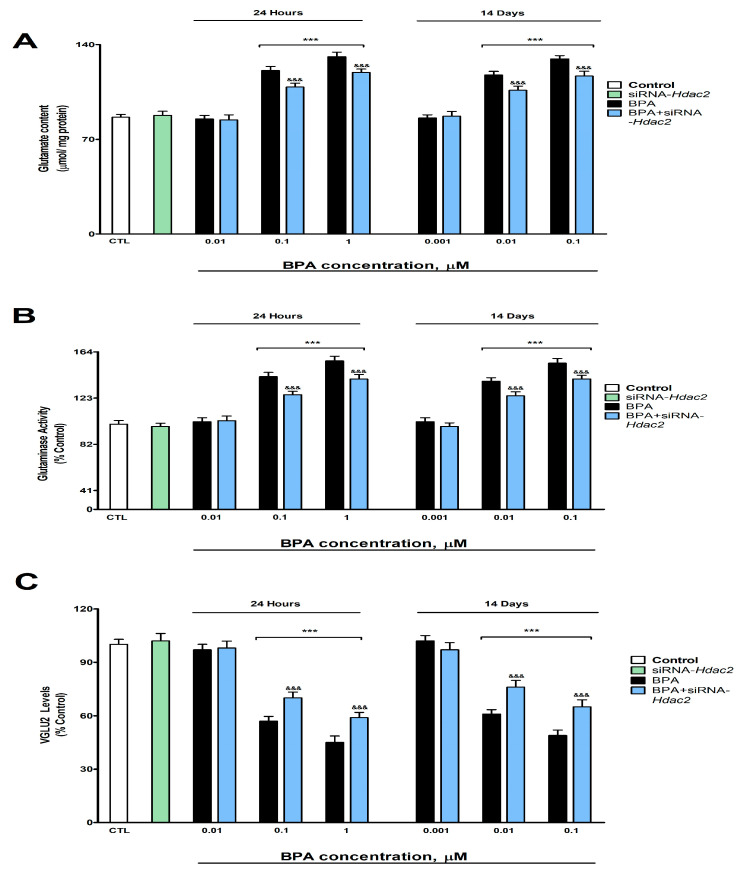
(**A**) Glutamate concentration, (**B**) glutaminase function, and (**C**) VGLUT2 levels. The absolute control value of VGLUT2 protein levels was 26.31 ± 0.87 ng/g. Results show the mean (n = 9) ± SEM of three independent experimental conditions from cells of different cultures, each developed in triplicate. There were no significant differences between controls treated with and without a vehicle, cells transfected without siRNA or with scramble siRNA, or cells transfected with or without siRNA and treated with a vehicle in any of the targets studied. The data shown in the figures represent control treated with vehicle and transfected without siRNA as 100%. One-way (treatment-response comparisons) and two-way (transfection/treatment comparisons) ANOVA analyses followed by Tukey post-hoc test were developed to determine statistically significant differences between treatments. *** *p* ≤ 0.001, significantly different from controls; ^&&&^ *p* ≤ 0.001 contrasted with BPA exposure.

BPA exposure during 1- (starting at 0.1 µM) or 14-days (starting at 0.01 µM) induced a concentration-dependent increase in glutamate content (Figure 2A) and glutaminase activity (Figure 2B), and a reduction in the VGLUT2 protein content (Figure 2C), corroborated by gene expression results (Supplementary Figure S2). BPA treatment (1- or 14-days) of SN56 cells transfected with siRNA against *Hdac2* reversed, in part, the increase in glutamate content and glutaminase activity and the decrease in VGLUT2 content, induced following BPA single exposure of not transfected cells (Figure 2). The *Hdac2* knockdown alone did not alter glutamate concentration, glutaminase activity, and VGLUT2 protein levels (Figure 2).

### 3.3. Analysis of Synaptic Proteins (PSD95, SPN, SYP, and NMDAR1 Protein Content and Gene Expression)

**Figure 3:**
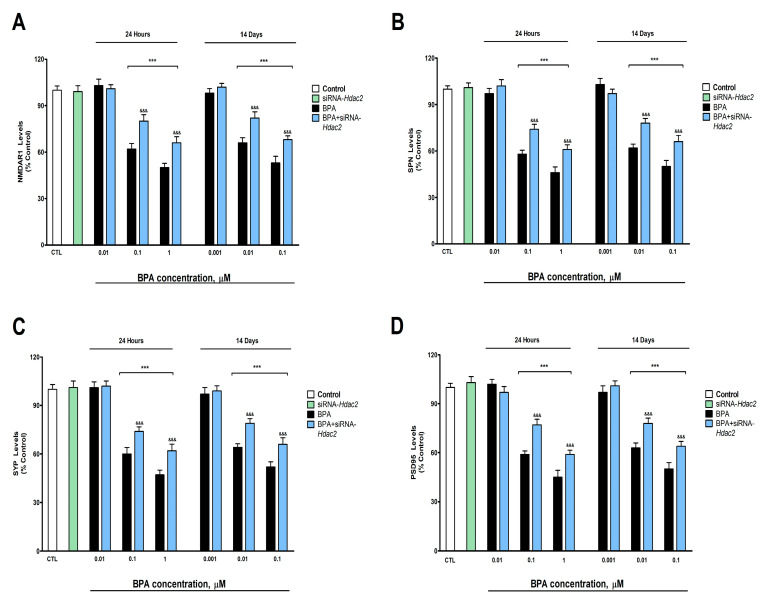
(**A**) NMDAR1, (**B**) SPN, (**C**) SYP, and (**D**) PSD95 levels. The absolute control values of NMDAR1, SPN, SYP, and PSD95 protein levels were 40.81 ± 1.31 ng/g, 26.12 ± 0.88 ng/g, 17.5 ± 0.57 ng/g, and 30.91 ± 1.05 ng/g, respectively. Results show the mean (n = 9) ± SEM of three independent experimental conditions from cells of different cultures, each developed in triplicate. There were no significant differences between controls treated with and without a vehicle, cells transfected without siRNA or with scramble siRNA, or cells transfected with or without siRNA and treated with a vehicle in any of the targets studied. The data shown in the figures represent control treated with vehicle and transfected without siRNA as 100%. One-way (treatment-response comparisons) and two-way (transfection/treatment comparisons) ANOVA analyses followed by Tukey post-hoc test were developed to determine statistically significant differences between treatments. *** *p* ≤ 0.001, significantly different from controls; ^&&&^ *p* ≤ 0.001 contrasted with BPA exposure.

BPA exposure during 1- (starting at 0.1 µM) or 14-days (starting at 0.01 µM) induced a concentration-dependent reduction in the NMDAR1 (Figure 3A), SPN (Figure 3B), SYP (Figure 3C), and PSD95 (Figure 3D) protein content, corroborated by gene expression results (Supplementary Figure S3). BPA treatment (1- or 14-days) of *Hdac2* silenced SN56 cells reversed, in part, the decrease in NMDAR1, PSD95, SPN, and SYP content, produced after BPA single treatment (Figure 3). The *Hdac2* silencing alone did not alter the concentration of the studied proteins (Figure 3).

### 3.4. Analysis of WNT/β-Catenin Pathway (GSK3β Activity, and pGSK3β (Ser 9), Cyclin D1, c-Myc and β-Catenin Protein Concentration and Gene Expression)

**Figure 4:**
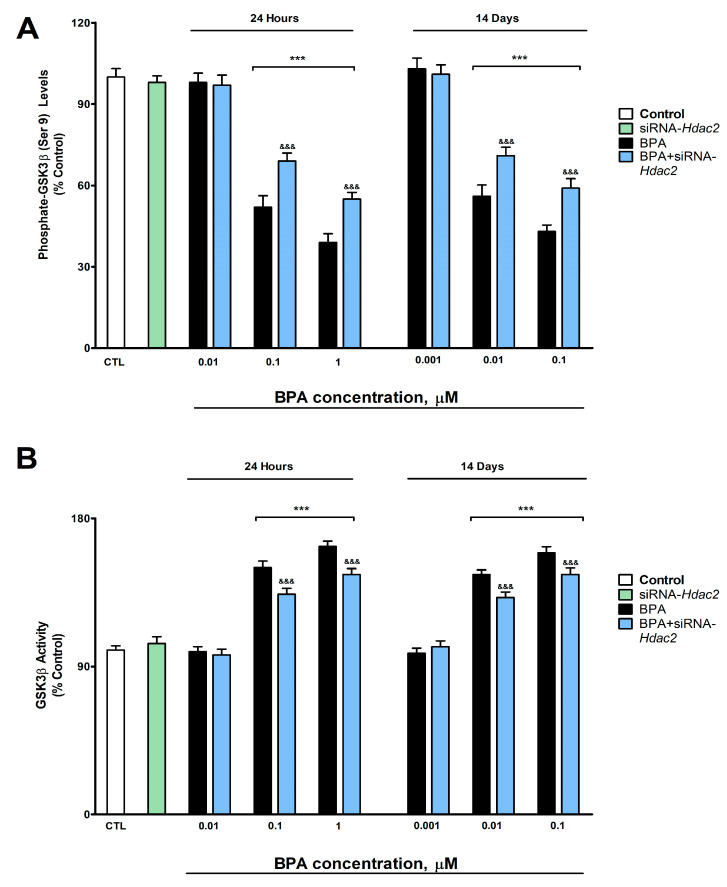
(**A**) p-GSK3β (Ser9) levels, and (**B**) GSK3β activity. Results show the mean (n = 9) ± SEM of three independent experimental conditions from cells of different cultures, each developed in triplicate. There were no significant differences between controls treated with and without a vehicle, cells transfected without siRNA or with scramble siRNA, or cells transfected with or without siRNA and treated with a vehicle in any of the targets studied. The data shown in the figures represent control treated with vehicle and transfected without siRNA as 100%. One-way (treatment-response comparisons) and two-way (transfection/treatment comparisons) ANOVA analyses followed by Tukey post-hoc test were developed to determine statistically significant differences between treatments. *** *p* ≤ 0.001, significantly different from controls; ^&&&^ *p* ≤ 0.001 contrasted with BPA exposure.

**Figure 5:**
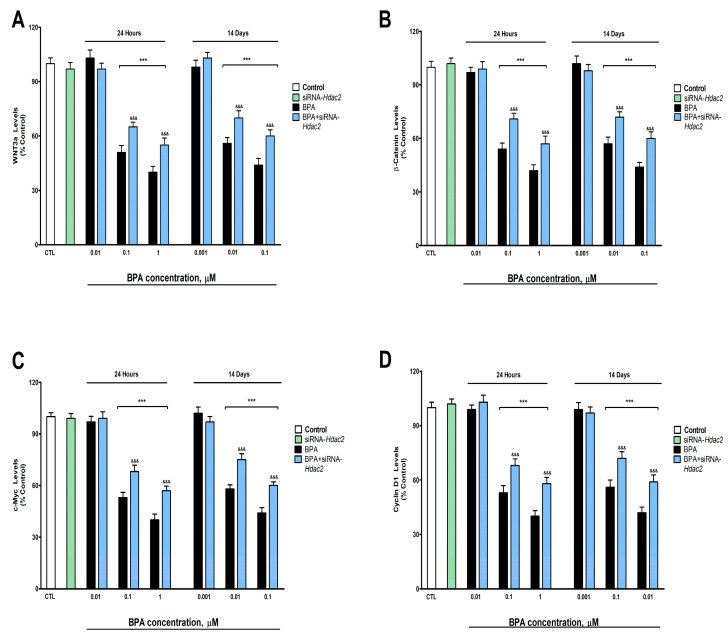
(**A**) WNT3a, (**B**) β-catenin, (**C**) c-Myc, and (**D**) cyclin D1 levels. The absolute control values of WNT3, β-catenin, c-Myc, and cyclin D1 protein content were 11.31 ± 0.21 ng/g, 22.14 ± 0.34 ng/g, 14.1 ± 0.52 ng/g, and 8.2 ± 0.31 ng/g, respectively. Results show the mean (n = 9) ± SEM of three independent experimental conditions from cells of different cultures, each developed in triplicate. There were no significant differences between controls treated with and without a vehicle, cells transfected without siRNA or with scramble siRNA, or cells transfected with or without siRNA and treated with a vehicle in any of the targets studied. The data shown in the figures represent control treated with vehicle and transfected without siRNA as 100%. One-way (treatment-response comparisons) and two-way (transfection/treatment comparisons) ANOVA analyses followed by Tukey post-hoc test were developed to determine statistically significant differences between treatments. *** *p* ≤ 0.001, significantly different from controls; ^&&&^ *p* ≤ 0.001 contrasted with BPA exposure.

BPA induced, after 1- (from 0.1 µM) or 14-days (from 0.01 µM) of treatment, a concentration-dependent reduction in p-GSK3β (ser9) (Figure 4A), WNT3a (Figure 5A), β-Catenin (Figure 5B), c-Myc (Figure 5C) and Cyclin D1 (Figure 5D). BPA treatment for 1- or 14-days also increased significantly the GSK3β activity (from 0.1 µM and 0.01 µM concentrations, respectively, Figure 4B). BPA treatment (1- or 14-days) of SN56 cells transfected with siRNA against *Hdac2* reversed, in part, the reduction in the expression and content of these target proteins, corroborated by gene expression results (Supplementary Figure S4), and the increase of the GSK3β activity induced following BPA alone exposure of non-transfected cells (Figure 4 and Figure 5). The *Hdac2* silencing alone did not modify the p-GSK3β (ser9) (Figure 4A), WNT3a (Figure 5A), β-Catenin (Figure 5B), c-Myc (Figure 5C) and Cyclin D1 (Figure 5D) proteins content, and the GSK3β activity (Figure 4B).

### 3.5. Gene Knockdown Evaluation

**Figure 6:**
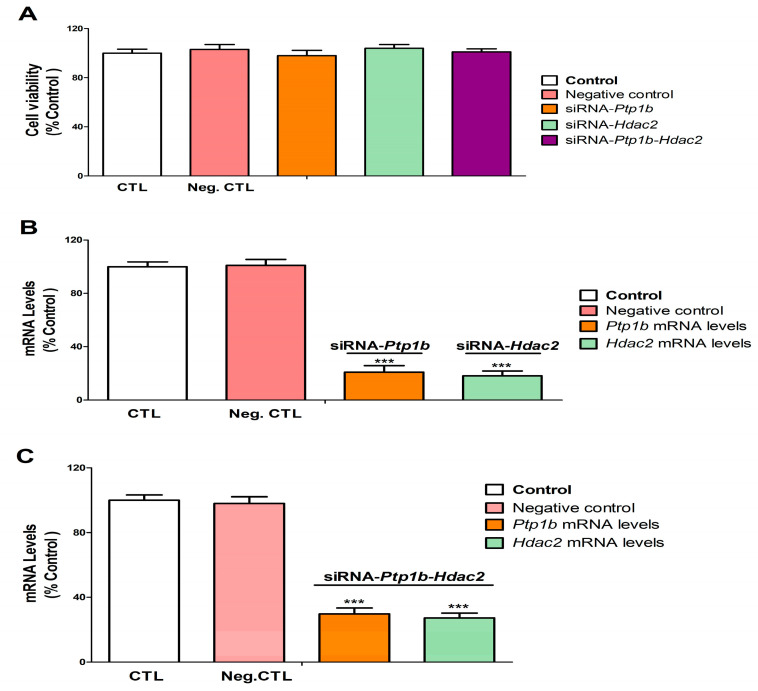
*Hdac2* and/or *Ptp1b* silencing efficiency and effect on cell viability. Positive and negative control (transfection without or with scrambled siRNA). *Hdac2*-siRNA, *Ptp1b*-siRNA, and *Ptp1b-Hdac2*-siRNA (transfection with siRNA against *Hdac2, Ptp1b, or Ptp1b-Hdac2*). The MTT absolute control value of absorbance at 562 nm was 0.80 ± 0.018. MTT analysis (**A**). *Hdac2* and *Ptp1b* gene expression analysis (**B**,**C**). Data show the mean (n = 9) ± SEM of three independent experiments from cells of different cultures, each developed in triplicate. There were no significant differences between controls treated with and without a vehicle, cells transfected without siRNA or with scramble siRNA, or cells transfected with or without siRNA and treated with a vehicle in any of the targets studied. The data shown in the figures represent control treated with vehicle and transfected without siRNA as 100%. One-way (treatment-response comparisons) and two-way (transfection/treatment comparisons) ANOVA analyses followed by Tukey post-hoc test were developed to determine statistically significant differences between treatments. *** *p* ≤ 0.001, significantly different from controls.

Transfection of SN56 cells with a negative control (NC), *Hdac2,* and/or *Ptp1b* siRNA alone had no effect on cell viability (Figure 6A). SN56 cells transfection with NC siRNA did not alter *Hdac2* and *Ptp1b* gene expression (Figure 6B,C). SN56 cells transfection with single or simultaneous *Hdac2* and *Ptp1b* siRNA alone significantly decreased *Hdac2* and *Ptp1b* gene expression (Figure 6B,C).

### 3.6. Analysis of the Effect of BPA on SN56 Cell Viability and Caspases 3/7 Activation

**Figure 7:**
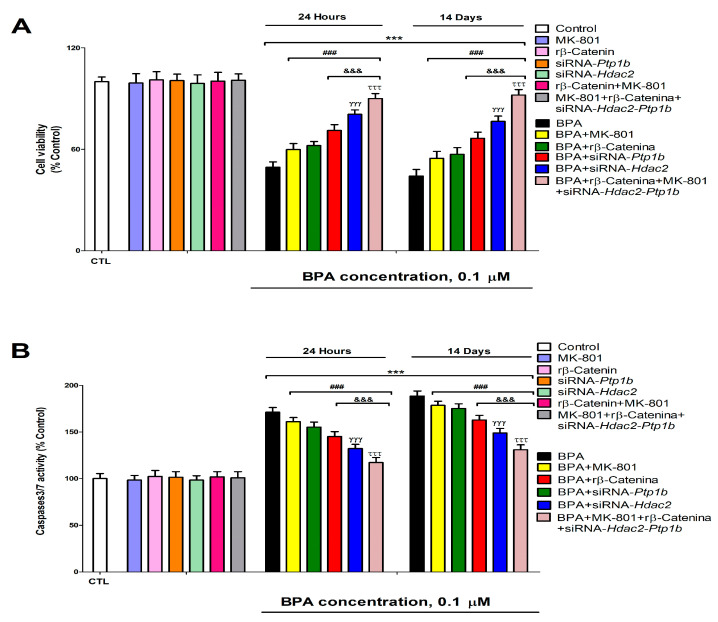
BPA (0. 1 µM) cell viability (**A**) and apoptosis (**B**) effects on transfected or non-transfected SN56 cells with siRNA against *Hdac2* and/or *Ptp1b* either alone or in combination with MK-801 (20 µM) and/or rβ-Catenin (15 µM) following 1- or 14-days. The data represent the mean (n = 9) ± SEM of three separate experiments from cells of different cultures, each of them performed in triplicate. There were no significant differences between controls treated with and without a vehicle, cells transfected without siRNA or with scramble siRNA, or cells transfected with or without siRNA and treated with a vehicle in any of the targets studied. The data shown in the figures represent control treated with vehicle and transfected without siRNA as 100%. One-way (treatment-response comparisons) and two-way (transfection/treatment comparisons) ANOVA analyses followed by Tukey post-hoc test were developed to determine statistically significant differences between treatments. *** *p* ≤ 0.001 compared to the control; ^###^ *p* ≤ 0.001 compared to BPA treatment; ^&&&^ *p* ≤ 0.001 compared to BPA co-treatment with rβ-Catenin; ^γγγ^ *p* ≤ 0.001 compared to BPA treatment of *Ptp1b* silenced cells; ^τττ^ *p* ≤ 0.001 compared to *Hdac2* knockdown cells exposed to BPA.

BPA induces, after 1- (from 0.1 µM) or 14-days (from 0.1 µM) of treatment, a significant reduction of SN56 cell viability (Figure 7A). The decrease observed in cellular survival following BPA exposure was partially reversed after co-exposure to MK-801 or rβ-Catenin, and BPA, or after exposure to BPA of transfected cells with *Hdac2* or *Ptp1b* siRNA alone (Figure 7A). There were no changes in cellular survival following single transfection with *Ptp1b* or *Hdac2* siRNA alone, single MK-801 or rβ-Catenin treatment, simultaneous MK-801 and rβ-Catenin co-treatment, or simultaneous co-exposure to MK-801 and rβ-Catenin of simultaneously transfected cells with *Ptp1b* and *Hdac2* siRNA (Figure 7A). Cellular survival decrease data did not significantly change when comparing BPA (0.1 µM) co-treatment with MK-801 or with rβ-Catenin (Figure 7A). BPA (0.1 µM) treatment of *Ptp1b* knockdown cells induced a lower significant decrease in cell survival compared to that observed after simultaneous BPA (0.1 µM) co-treatment with MK-801 or with rβ-Catenin (Figure 7A). Exposure to BPA (0.1 µM) of *Hdac2* knockdown cells led to a lower significant reduction of cellular survival than that observed following exposure to BPA (0.1 µM) of *Ptp1b* knockdown cells (Figure 7A). Concomitant BPA (0.1 µM), MK-801, and rβ-Catenin co-treatment of cells transfected with *Hdac2* and *Ptp1b* siRNA led to the highest reversion of cellular survival decrease observed after exposure to BPA of non-transfected cells (Figure 7A). Results observed on control cells and vehicle treatment showed no significant variance.

The activity of caspases 3/7 was analyzed to determine apoptosis induction. BPA significantly increased, after 1- (from 0.1 µM) or 14-days (from 0.1 µM) of treatment, caspases 3/7 activity in SN56 cells (Figure 7B). Caspases 3/7 activity increase observed following exposure to BPA was partially reversed by co-exposure to MK-801 or rβ-Catenin, and BPA or after exposure to BPA of cells transfected with *Hdac2* or *Ptp1b* siRNA (Figure 7B). There was no effect on caspases 3/7 activation following a single knockdown of *Ptp1b* or *Hdac2* alone, single MK-801 or rβ-Catenin treatment, simultaneous MK-801, and rβ-Catenin co-treatment, or simultaneous co-exposure to MK-801 and rβ-Catenin of simultaneously transfected cells with *Ptp1b* and *Hdac2* siRNA (Figure 7B). Caspases 3/7 activity increase data did not significantly change when comparing BPA (0.1 µM) co-treatment with MK-801 or with rβ-Catenin (Figure 7B). BPA (0.1 µM) treatment of *Ptp1b* knockdown cells induced a lower significant increase of caspases 3/7 activity compared to observed after simultaneous BPA (0.1 µM) co-treatment with MK-801 or with rβ-Catenin (Figure 7B). Exposure to BPA (0.1 µM) of *Hdac2* knockdown cells led to a lower significant increase of caspases 3/7 activity than that observed following exposure to BPA (0.1 µM) of *Ptp1b* knockdown cells (Figure 7B). Concomitant BPA (0.1 µM), MK-801, and rβ-Catenin co-treatment of cells transfected with *Hdac2* and *Ptp1b* siRNA led to the highest reversion of caspases 3/7 activity increase observed after exposure to BPA of non-transfected cells (Figure 7B). Results observed on control cells and vehicle treatment showed no significant variance. Results show that exposure to BPA led to apoptosis, which corroborates cellular survival data.

## 4. Discussion

Data obtained in the present research is the first to point out that single BPA (40 µg/kg) treatment, after 2 days, increases the percentage of necrotic (ChAT− and ChAT+) neurons in BF. Cholinergic neurons appear to be more sensitive to BPA, as a higher increase in the percentage of necrotic neurons was observed in ChAT+ neurons. To our knowledge, no previous studies have determined the BPA histological effects on BF. Previous studies described neurodegeneration in rat hippocampal and cortical neurons after BPA repeated oral exposure during adulthood [49,50,51]. Additionally, single BPA (1 mg/kg) intraperitoneal treatment was shown to induce cellular inflammation, edema, and necrosis in adult rat hippocampal neurons [52]. These previous works support our present data.

The amount of neurons (NeuN+ or ChAT+) was not significantly decreased following BPA treatment alone, despite the fact that there was a significant increase in necrotic neurons (ChAT− and ChAT+) in BF, which suggests that the dose chosen for a single treatment administration was insufficient to induce extensive damage and a significant loss of all different cell types, but enough to induce selective neuronal damage. To our knowledge, previous studies describing necrosis induction after single and repeated BPA exposure in adult rats did not examine neuronal density, so further studies on BPA effects after single and repeated treatment on different types of brain cells population density in adulthood are needed, to better characterize the damage induced by this compound on the brain and the mechanisms that mediate such damage.

Otherwise, exposure to BPA during one- (starting at 0.1 µM) or 14-days (starting at 0.01 µM) led to an increase in the glutamate concentration and the glutaminase activity, and a reduction in the VGLUT2, NMDAR1, SPN, SYP, and PSD95 protein content, pointing out synaptic plasticity proteins regulators and glutamate neurotransmission disruption. VGLUT2, highly expressed in BF, mediates the uptake of synaptic glutamate, preventing its accumulation in the synaptic cleft [53]. Previous studies have shown that BPA increases glutamate content in rat hippocampus and frontal cortex after continuous exposure [13] and in mice hippocampus after postnatal exposure, through the decrease of glutamate uptake [14], supporting our results. However, BPA was also reported to reduce the content of glutamate by decreasing the activity of glutaminase, which mediates the synthesis of glutamate [15]. These contradictions could be due to differences in the length of the exposure period, the doses used, or the developmental exposure period chosen. BPA was described to decrease dendritic spine density through synaptic proteins NMDAR1, PSD95, and synapsin I downregulation, leading to alterations in synaptic plasticity regulation and, finally, cognitive dysfunction in adult mice hippocampus following long-term exposure [2]. Acute exposure to BPA also resulted in reduced dendritic spine density, PSD95 and NMDA receptors expression, and cognitive decline [1]. BPA prenatal exposure was also reported to decrease synaptic plasticity and the gene and protein expression of NDMAR1, SYP, SPN, and PSD95, pointing out the ability of BPA to rule their expression, leading to alterations in dendritic spines and the induction of cognitive decline [11]. Thus, the BPA glutamatergic transmission and synaptic protein disruption shown in SN56 cells could mediate synaptic plasticity dysfunction in BFCN, if corroborated in vivo, leading to the cognitive decline described.

Exposure to BPA during one- (starting at 0.1 µM) or 14-days (starting at 0.01 µM) of SN56 transfected with siRNA against *Hdac2* reversed, in part, the increase in the glutamate content and the glutaminase activity, and the decrease in VGLUT2, NMDAR1, PSD95, SPN, and SYP protein content observed following exposure to BPA of non-transfected cells. BPA was also described to decrease dendritic spine density through *Hdac2* overexpression after repeated exposure and its inhibition reverts this effect [10]. Capsaicin-induced HDAC2 protein overexpression was reported to reduce dendritic spine density through the downregulation of synaptic proteins NDMAR1, SYP, SPN, and PSD95, among others, in mice primary hippocampal neurons [19], supporting our findings. Although no studies were performed to determine HDAC2 effects on glutaminase activity, paclitaxel-induced HDAC2 protein expression was reported to regulate VGLUT2 and glutamate levels, and its silencing to decrease glutamate levels, VGLUT2 and SPN protein expression in rat spinal dorsal horn, supporting our results [20]. Thus, although BPA alters these targets through HDAC2 protein overexpression, which could mediate the synaptic plasticity and cognitive dysfunctions previously described, other mechanisms seem to be involved. BPA has been reported to induce HDAC2 protein expression, which mediates, partially, the disruption of the insulin signaling pathway [5]; being, the insulin signaling pathway, involved in the regulation of glutamate levels through glutamate release [54] and glutaminase activity modulation [55]. Additionally, insulin signaling disruption was reported to reduce SYP, PSD95, and NMADR1 protein expression [56,57] and to disrupt the NGF/P75^NTR^/TrkA pathway [4]. The latter was described to disrupt glutamate transmission, synaptic proteins, and plasticity [58,59,60]. Hence, the commented actions may play a role in the BPA harmful results described.

BPA induces, after 1- (from 0.1 µM) or 14-day (from 0.01 µM) of treatment, a concentration-dependent reduction in WNT3a, β-Catenin, Cyclin D1, p-GSK3β (Ser9), and c-Myc protein content, and an increase in GSK3β activity. Phosphorylation of β-Catenin leads to its degradation by proteasome and autophagy [61]. GSK3β phosphorylation in Ser9 residue, the main regulatory residue of GSK3β activity, leads to its inactivation [62]. The canonical WNT agonists, such as WNT3a, inactivate several kinases, including GSK3β, reducing β-Catenin phosphorylation, and its degradation, leading to its accumulation and induction of the transcription of WNT/β-Catenin pathway downstream target genes, as Cyclin D1 and c-Myc [63]. Therefore, the effect observed points out that BPA downregulates the WNT/β-Catenin pathway, probably by reducing WNT3a, which could activate GSK3β due to p-GSK3β (Ser9) decrease, leading to a β-Catenin, and the target genes regulated by it, decrease. BPA was reported to reduce β-Catenin, p-GSK-3β, and Cyclin-D1 protein content in neural stem cells [16], supporting our results. In contrast, BPA was shown to increase β-Catenin, WNT3a, and c-Myc protein expression in the submandibular salivary glands of rats, showing the BPA alteration of WNT/β-Catenin pathway through an increase in the production of the WNT3a [64], which supports the BPA ability to alter the WNT/β-Catenin pathway but in the opposite direction. These discrepancies may result from various factors including, but not limited to, the model used (in vivo vs. in vitro), tissues analyzed, or treatment schedule. Moreover, BPA treatment (1- or 14-days) of SN56 cells transfected with siRNA against *Hdac2* reversed, in part, the increase in GSK3β activity and the reduction in the expression and content of these target proteins, indicating that this mechanism mediates the effects observed, but other mechanisms could be involved. To our knowledge, this is the first time that BPA was shown to disrupt the WNT/β-Catenin pathway through HDAC2 protein overexpression. HDAC2 inhibition was reported to upregulate the WNT/β-Catenin pathway, supporting our findings [20]. Insulin and NGF/P75^NTR^/TrkA pathways were described to regulate WNT/β-Catenin pathway expression [65,66], so both mechanisms could also contribute to these alterations.

Otherwise, BPA-induced SN56 cell loss after 1- or 14-days of treatment (0.1 µM). This action was reversed after single concomitant exposure to MK-801, rβ-Catenin, and BPA, or after exposure to BPA of cells transfected with *Hdac2* or *Ptp1b* siRNA. These results point out that the processes described above play a role in the cell demise reported. We previously showed that HDAC2 upregulation and insulin pathway disruption through PTP1B protein overexpression mediates the SN56 cell loss induced by BPA following single and repeated treatment [5]. The WNT/β-Catenin pathway was reported to maintain cell viability and its downregulation induces cell death in rat BFCN and learning/memory alterations [17,18]. Excitotoxicity induced by glutamate accumulation has also been described to induce SN56 cell death [4], supporting, all these data, in our findings.

Finally, concomitant exposure to BPA, MK-801, and rβ-Catenin of cells transfected with *Hdac2* and *Ptp1b* siRNA induced the highest reversion of the cell demise produced following exposure to BPA of non-transfected cells, but it was still not complete, pointing out that further mechanisms may play a role. We described, in a previous work, that BPA single and repeated treatment upregulates P75^NTR^ protein expression and increases the Aβ proteins accumulation in SN56 cells, which were reported to bind to P75^NTR^ triggering cell death [5]. BPA was also shown to alter thyroid hormone pathways, which are necessary to avoid BFCN loss, and their disruption leads to BFCN loss [5]. Therefore, these mechanisms, as well as those presented above, and those previously described could mediate the cell death observed in SN56 cholinergic neurons from BF, which, if corroborated in vivo, may be able to mediate the cognitive dysfunction reported after BPA exposure.

## 5. Conclusions

In summary, BPA produced a more pronounced percentage of cholinergic necrotic neurons than of other types of neurons in the BF after a single treatment in rats, and induced, in SN56 cells from BF, synaptic proteins (SPN, SYP, and PSD95) downregulation and glutamate transmission disruption through VGLUT2 and NMDAR1 downregulation, and glutaminase activity decrease after single and repeated treatment. BPA also induced WNT/β-Catenin pathway downregulation and triggered cell death in SN56 cholinergic neurons from BF after 1- or 14-days of treatment. All BPA toxic effects in SN56 cells were mediated, in part, by HDAC2 upregulation. Further research is needed to unravel the remaining processes that mediate the BPA cytotoxic effects described in SN56 cells, which should be corroborated in vivo, together with those previously shown to mediate these effects, verifying if they produce the cognitive decline induced by BPA. Our results provide new information on the higher sensitivity of BFCN to BPA toxic effects, the mechanisms that mediate the WNT/β-Catenin pathway and synaptic plasticity disruption, and the cytotoxic mechanism that mediates SN56 cholinergic cell death. The corroboration in vivo of the BPA toxic mechanisms described previously, and above, in SN56 cells could explain the neurotoxic effect in BFCN in vivo and the subsequent neurodegeneration induced in the hippocampus and cortex and the cognitive decline produced after BPA exposure, which could help to prevent and treat these toxic effects.

## Data Availability

Not applicable.

## Supplementary Materials

The following supporting information can be downloaded at: https://www.mdpi.com/article/10.3390/biology12060782/s1, Figure S1:BPA effects on Wistar rats’ diagonal band of Broca and Medial Septal nucleus NeuN+, ChAT+, and ChAT-/ChAT+ necrotic neurons after acute (40 µg/kg) exposure (Figure A–F); Figure S2: Vglut2 mRNA levels; Figure S3: Nmdar1, Spn, Syp and Psd95 mRNA levels; Figure S4: Wnt3a, β-catenin, c-Myc, and cyclin D1 mRNA levels.
