# Supplementary material for: Bisphenol-A Neurotoxic Effects on Basal Forebrain Cholinergic Neurons In Vitro and In Vivo
Source: Biology (Basel). 2023 May 28;12(6):782. doi: 10.3390/biology12060782 (PMC10294797; doi:10.3390/biology12060782)
Supplement: Supplementary file 1 [file biology-12-00782-s001.zip › SF1.pdf]

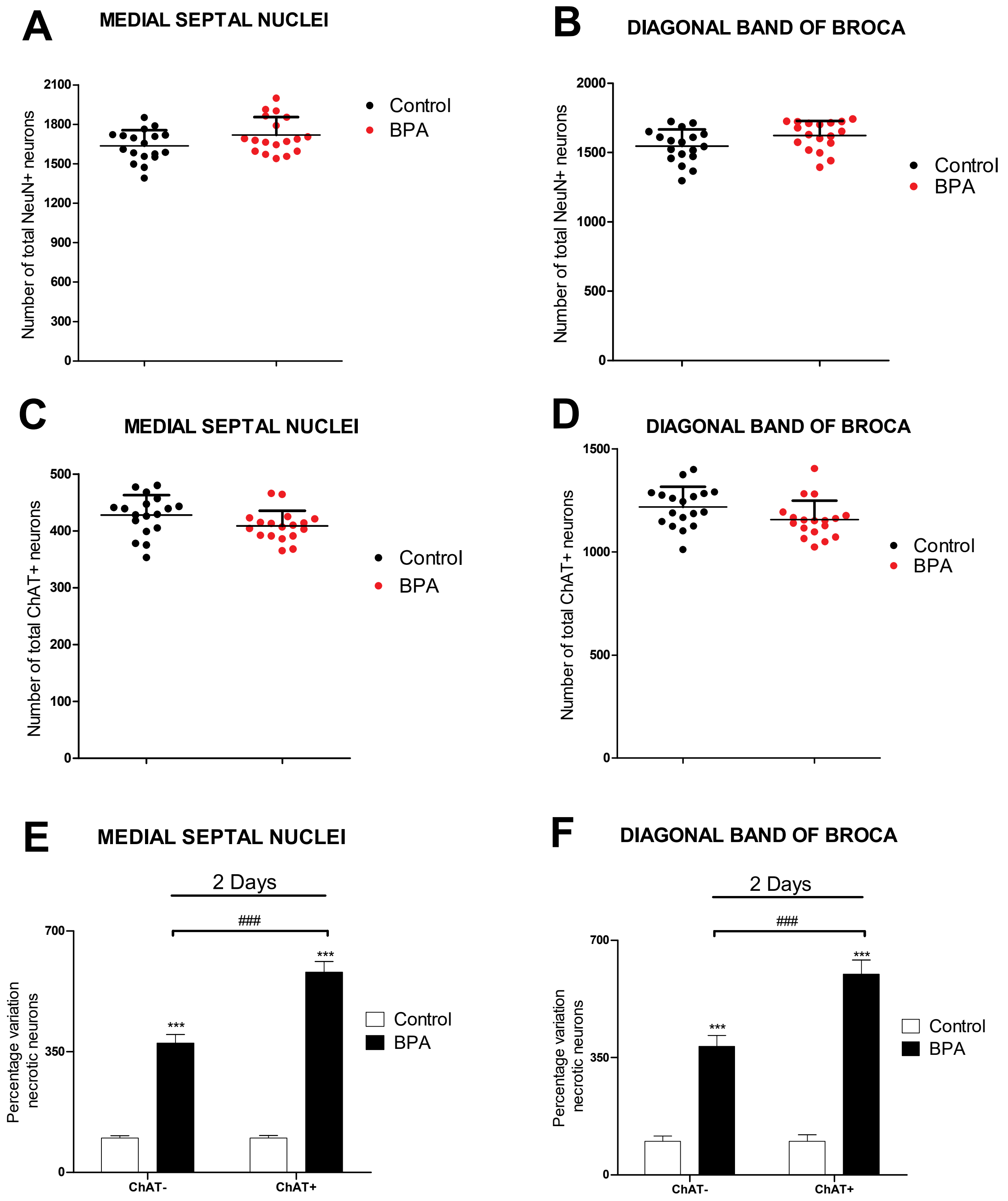
A
MEDIAL SEPTAL NUCLEI
Number of total NeuN+ neurons
2100
1800
1500
1200
900
600
300
0
Control
BPA
B
DIAGONAL BAND OF BROCA
Number of total NeuN+ neurons
2000
1500
1000
500
0
Control
BPA
C
MEDIAL SEPTAL NUCLEI
Number of total ChAT+ neurons
500
400
300
200
100
0
Control
BPA
D
DIAGONAL BAND OF BROCA
Number of total ChAT+ neurons
1500
1000
500
0
Control
BPA
E
MEDIAL SEPTAL NUCLEI
2 Days
###
***
***
Percentage variation necrotic neurons
700
350
0
ChAT-
ChAT+
Control
BPA
F
DIAGONAL BAND OF BROCA
2 Days
###
***
***
Percentage variation necrotic neurons
700
350
0
ChAT-
ChAT+
Control
BPA
