# Supplementary material for: Bisphenol-A Neurotoxic Effects on Basal Forebrain Cholinergic Neurons In Vitro and In Vivo
Source: Biology (Basel). 2023 May 28;12(6):782. doi: 10.3390/biology12060782 (PMC10294797; doi:10.3390/biology12060782)
Supplement: Supplementary file 1 [file biology-12-00782-s001.zip › Supplementary Figures Legends 3.pdf]

Supplementary Figures Legends.

Supplementary Figure S1. BPA effects on Wistar rats' diagonal band of Broca and Medial Septal nucleus NeuN+, ChAT+, and ChAT-/ChAT+ necrotic neurons after acute (40 µg/kg) exposure (figures A-F). Data represent the mean (n=6 animals per treatment) ± SD of the number of total NeuN neurons (figures A and B), total ChAT+ neurons (figures C and D) and the percentage variation of ChAT-/ChAT+ necrotic neurons (figures E and F) compared to control. Three sections were used per nuclei. At least 3 replicates of each type of neuron counting were performed. Two way-ANOVA (treatment vs type of neuron comparison) or Student's t-test (control vs treatment comparison). $^{***}p<0.001$ compared to control (figures E and F); $^{\#\#\#}p<0.001$ compared to ChAT- necrotic neurons (figures E and F).

Supplementary Figure S2. *Vglut2* mRNA levels. Data represent the mean (n=9) ± SEM of three separate experiments from cells of different cultures, each performed in triplicate. One-way (treatment-response comparisons) followed by Tukey posthoc test was developed to determine statistically significant differences between treatments. $^{***}p\leq0.001$, significantly different from controls; $^{\&\&\&}p\leq0.001$ compared to BPA treatment.

Supplementary Figure S3. (A) *Nmdar1*, (B) *Spn*, (C) *Syp* and (D) *Psd95* mRNA levels. Data represent the mean (n=9) ± SEM of three separate experiments from cells of different cultures, each performed in triplicate. One-way (treatment-response comparisons) followed by Tukey posthoc test was developed to determine statistically significant differences between treatments. $^{***}p\leq0.001$, significantly different from controls; $^{\&\&\&}p\leq0.001$ compared to BPA treatment.

Supplementary Figure S4. (A) *Wnt3a*, (B) *β-catenin*, (C) *c-Myc*, and (D) *cyclin D1* mRNA levels. Data represent the mean (n=9) ± SEM of three separate experiments from cells of different cultures, each performed in triplicate. One-way (treatment-response comparisons) followed by Tukey posthoc test was developed to determine statistically significant differences between treatments. $^{***}p\leq0.001$, significantly different from controls; $^{\&\&\&}p\leq0.001$ compared to BPA treatment.
