# Supplementary figures and images for: Bisphenol-A Neurotoxic Effects on Basal Forebrain Cholinergic Neurons In Vitro and In Vivo
Source: Biology (Basel). 2023 May 28;12(6):782. doi: 10.3390/biology12060782 (PMC10294797; doi:10.3390/biology12060782)

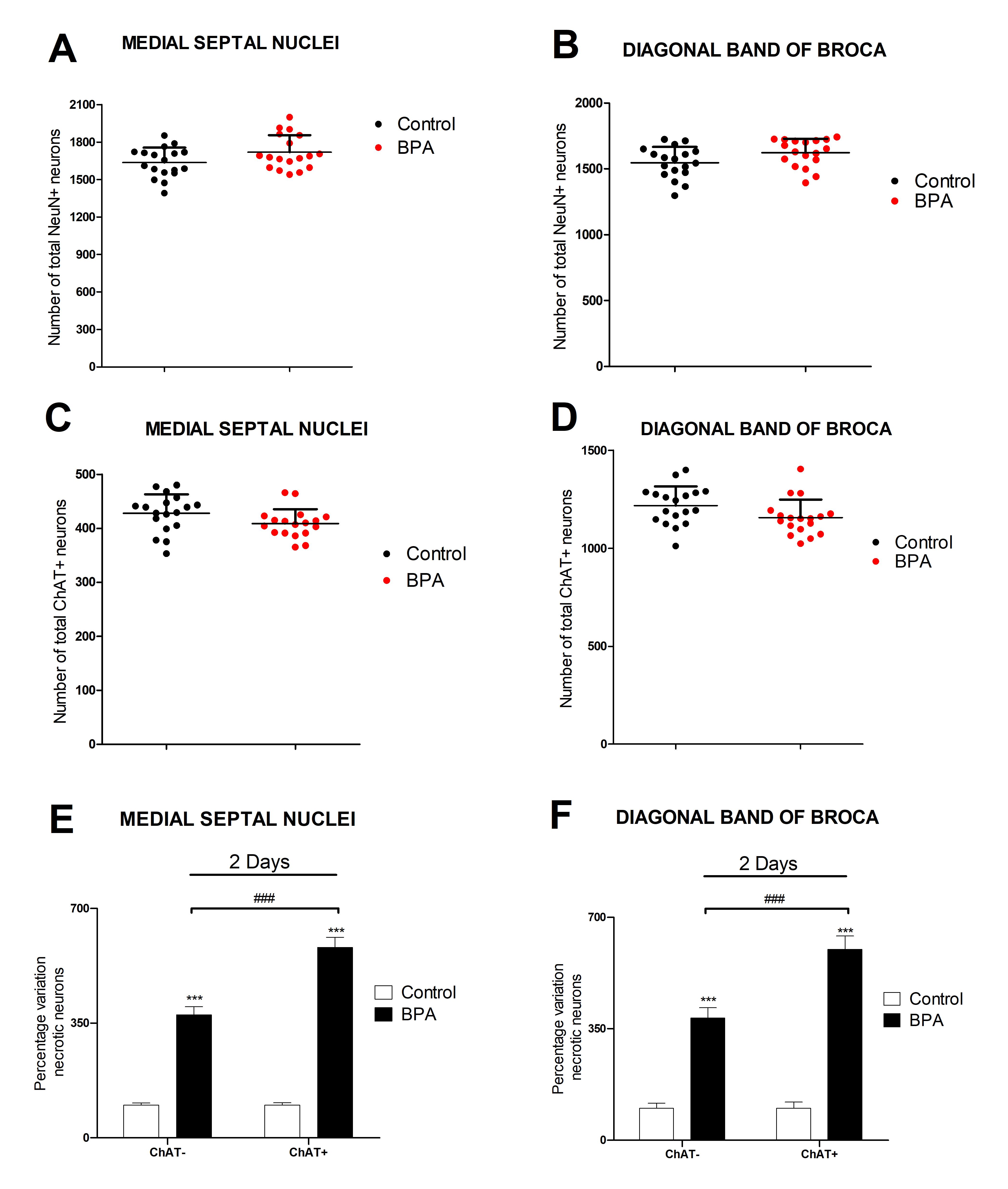

Supplement: Supplementary file 1 [file biology-12-00782-s001.zip › SF1.jpg]

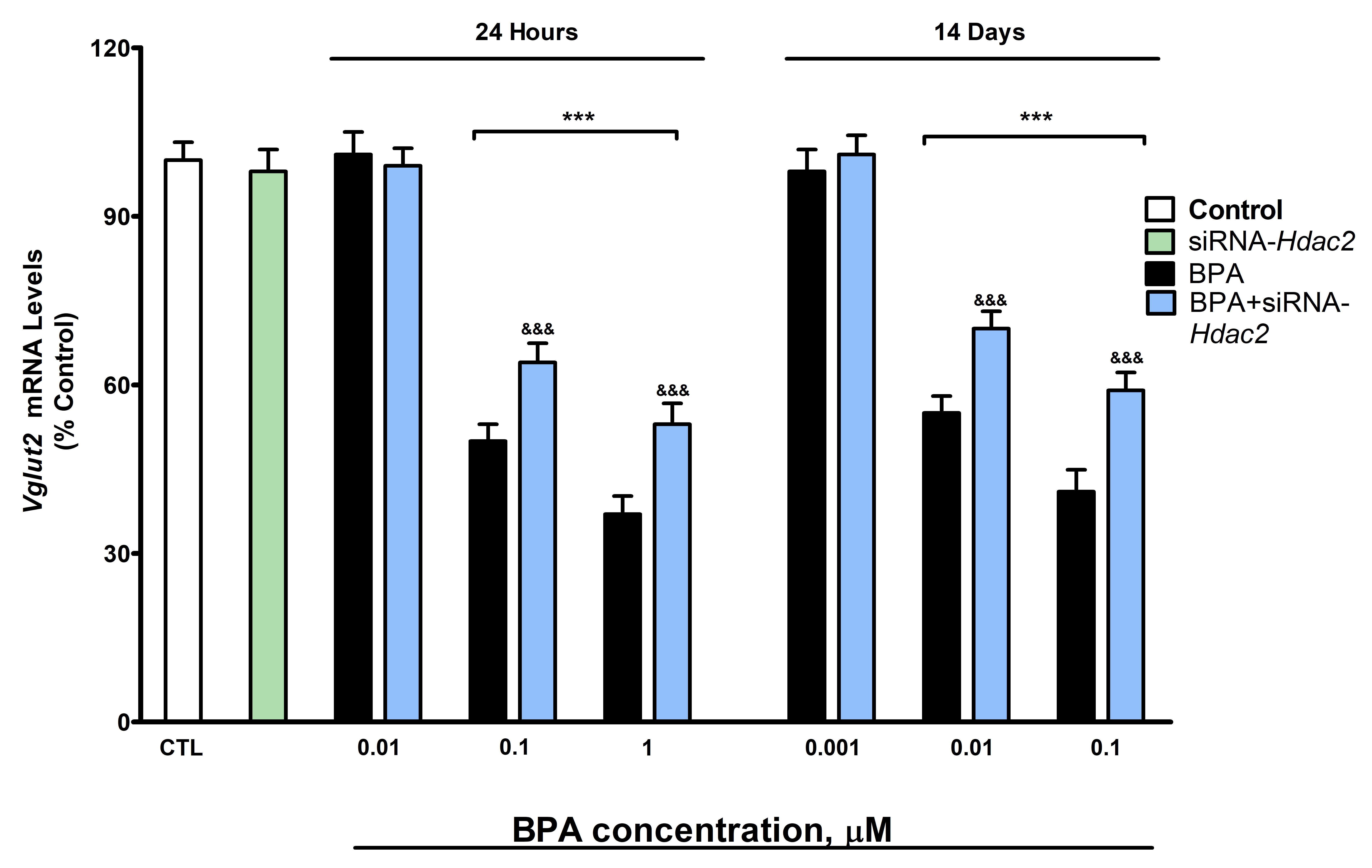

Supplement: Supplementary file 1 [file biology-12-00782-s001.zip › SF2.jpg]

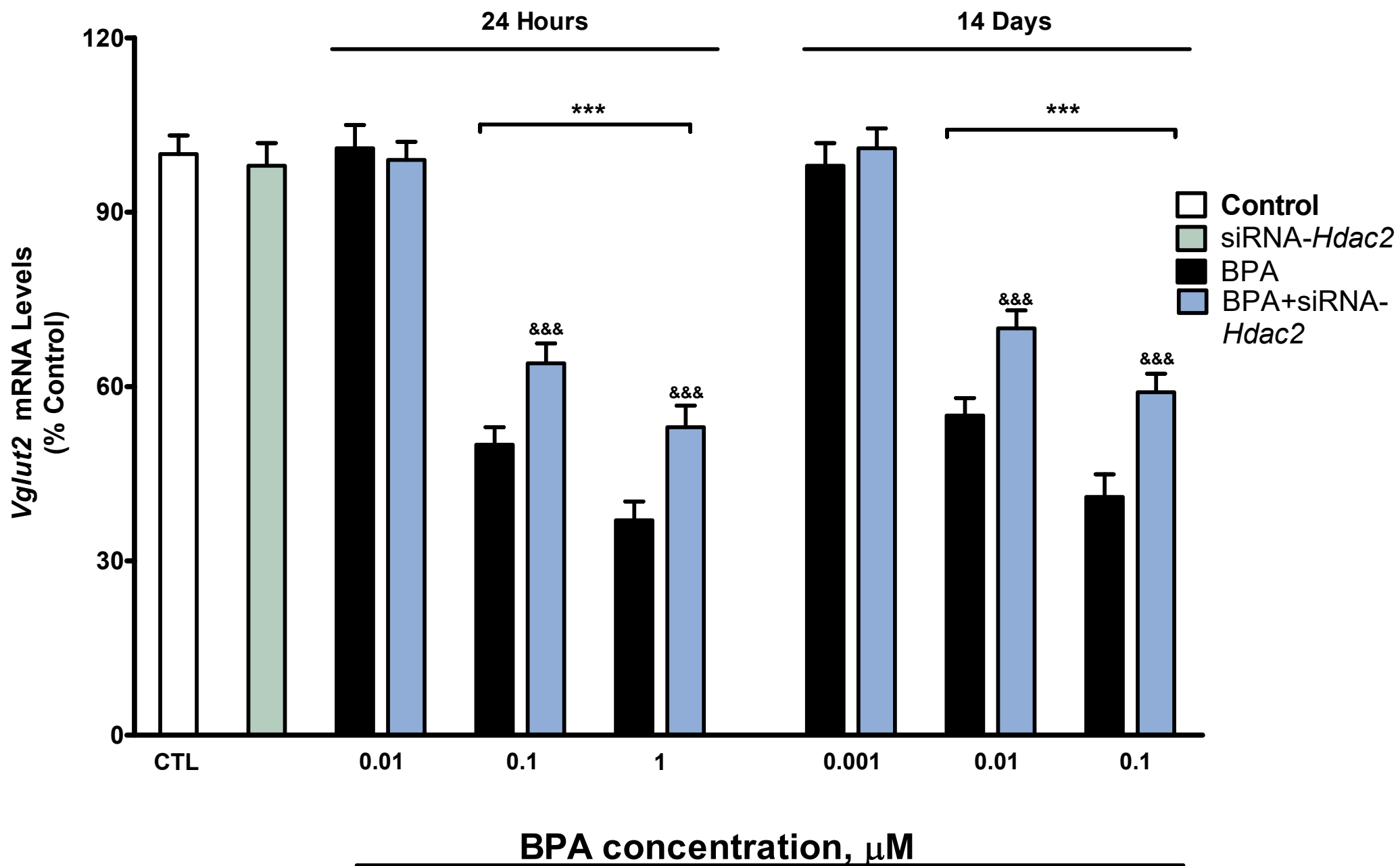

Supplement: Supplementary file 1 [file biology-12-00782-s001.zip › SF2.pdf]

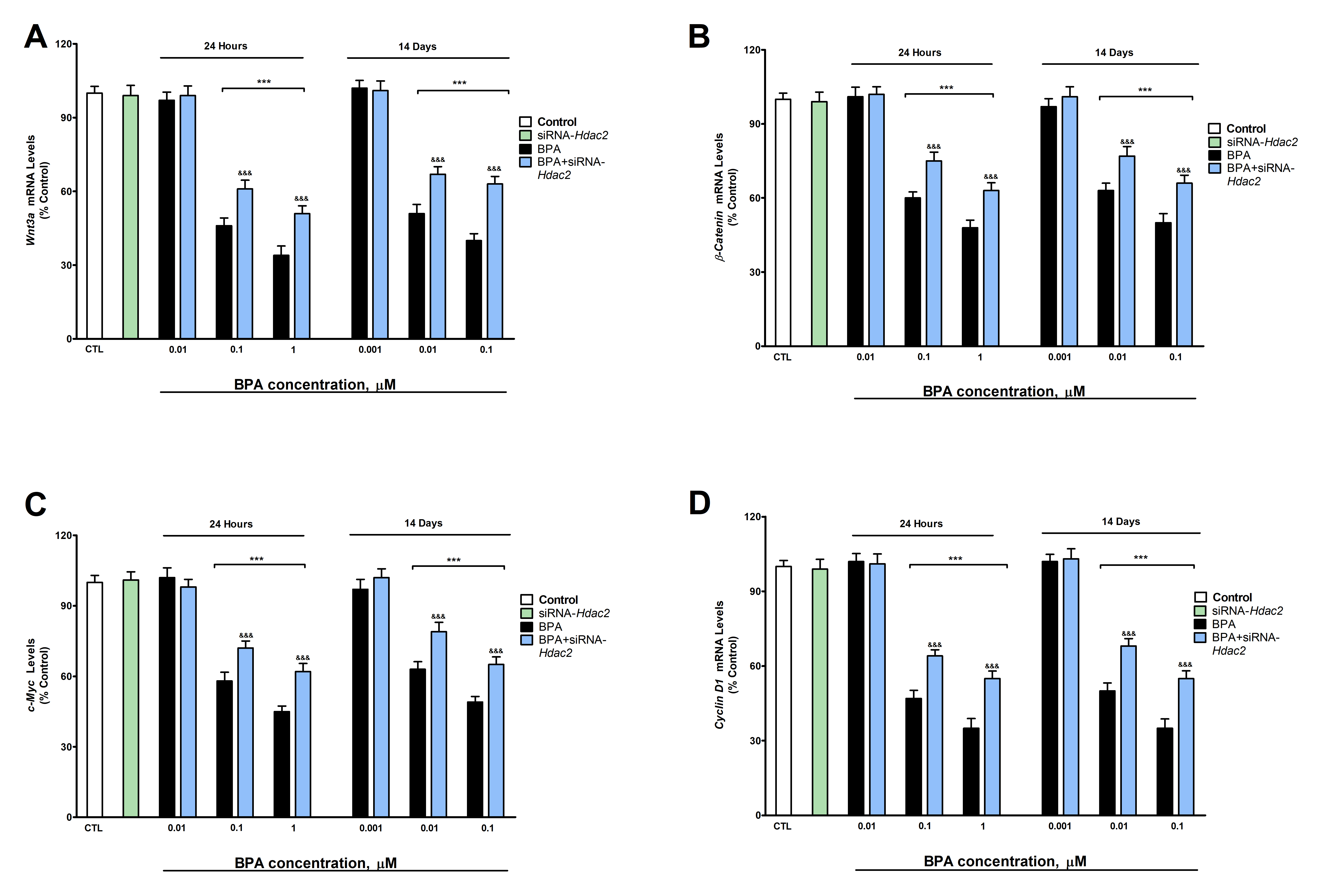

Supplement: Supplementary file 1 [file biology-12-00782-s001.zip › SF3.jpg]

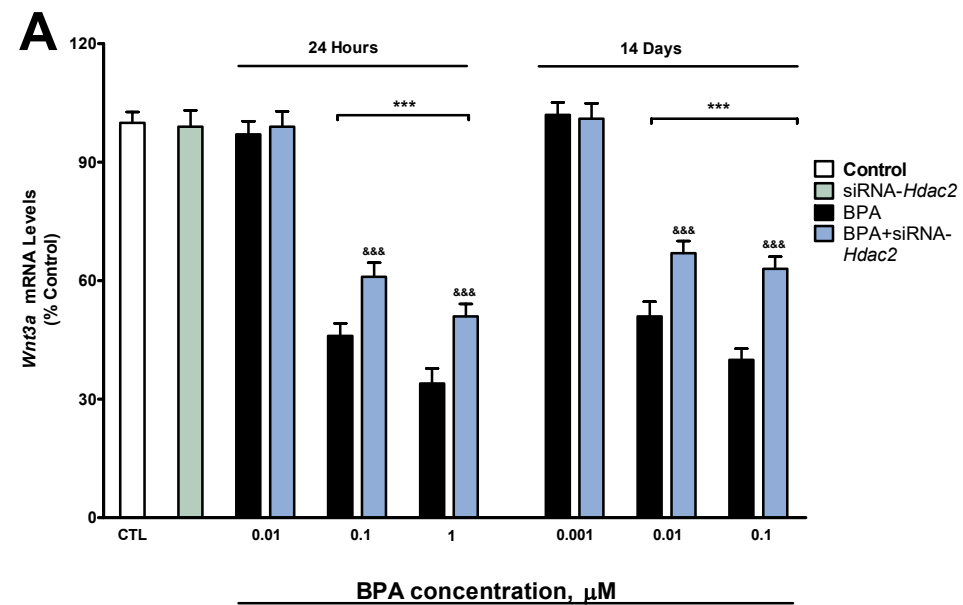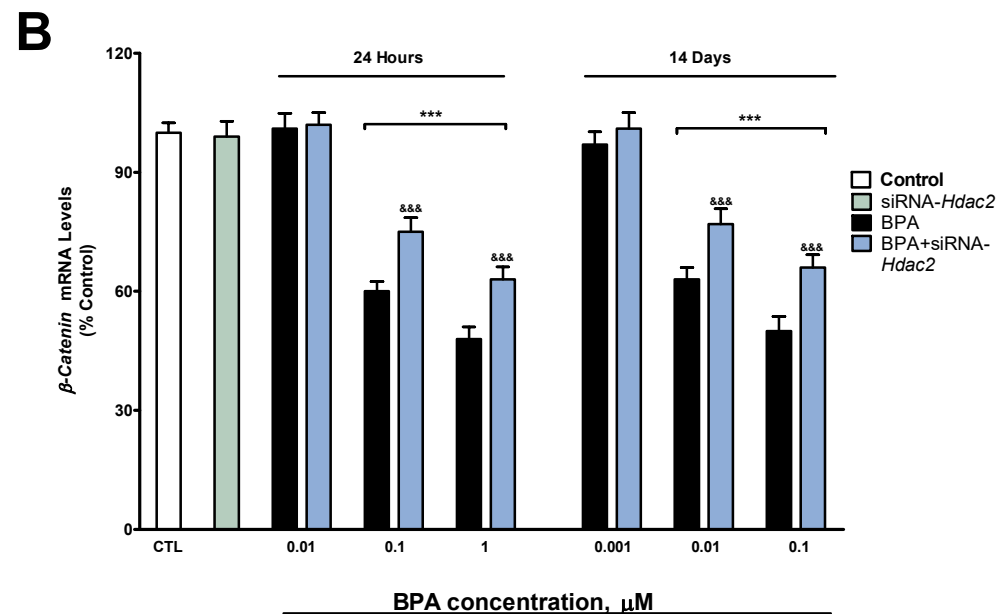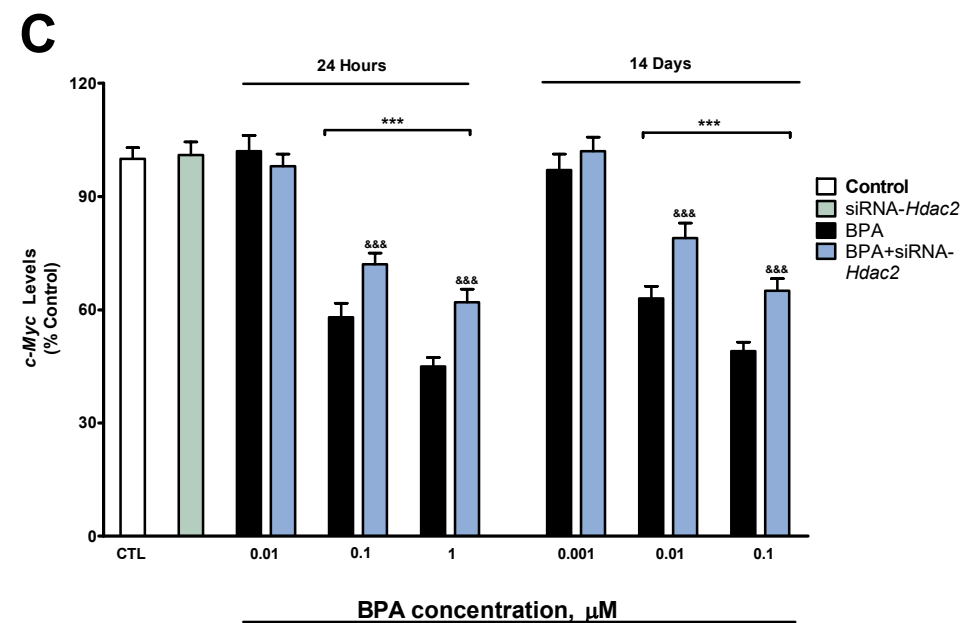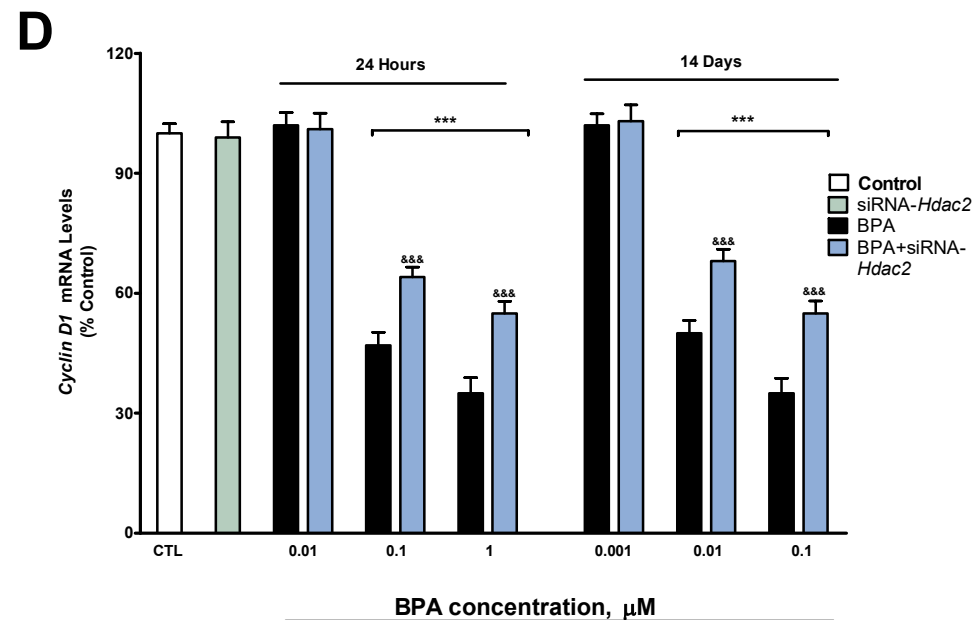

Supplement: Supplementary file 1 [file biology-12-00782-s001.zip › SF3.pdf]

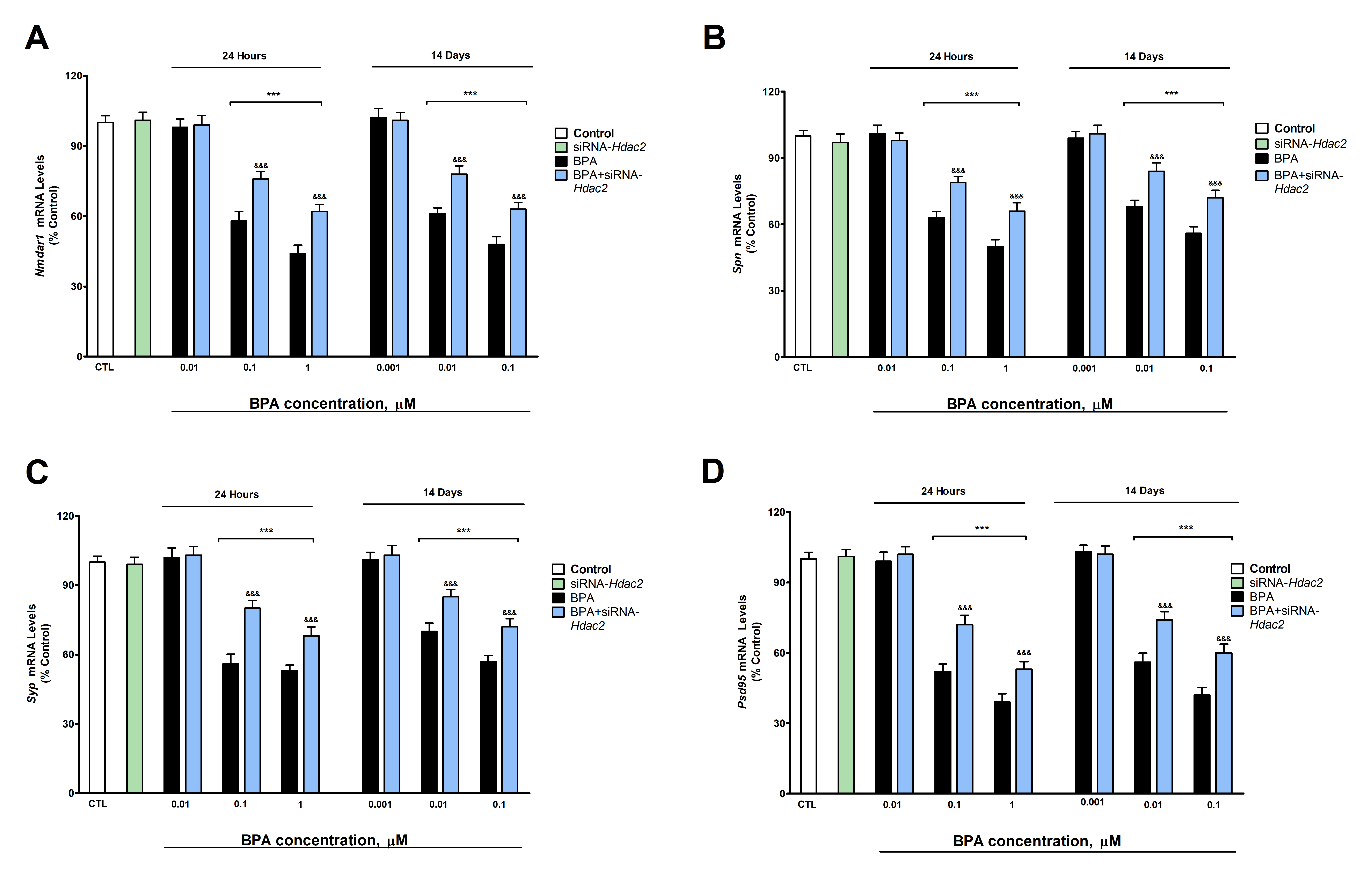

Supplement: Supplementary file 1 [file biology-12-00782-s001.zip › SF4.jpg]

**A**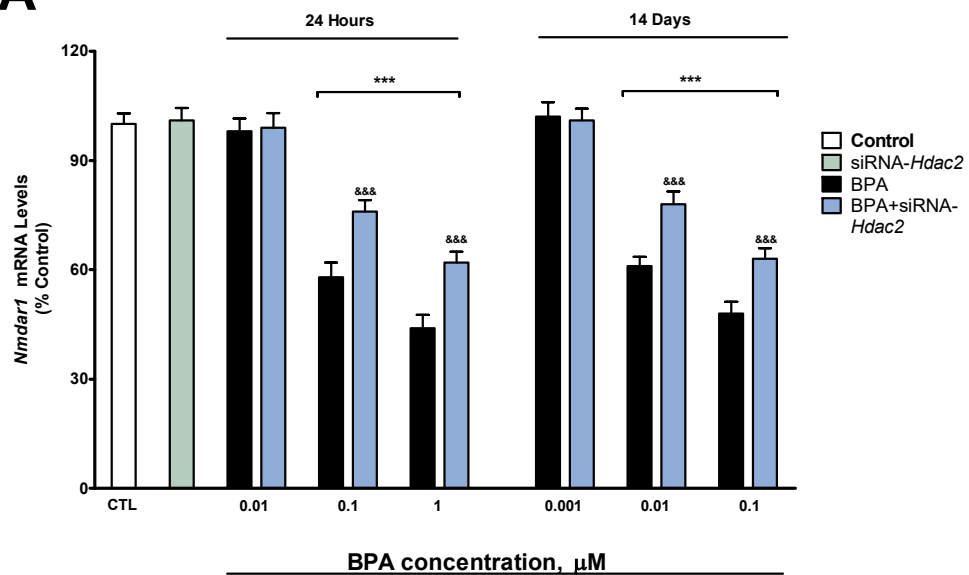**B**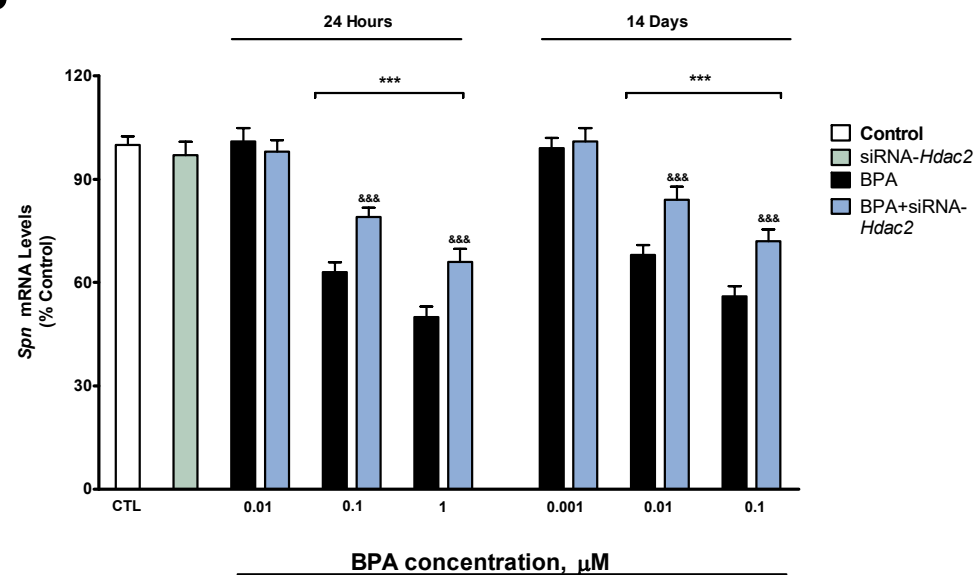**C**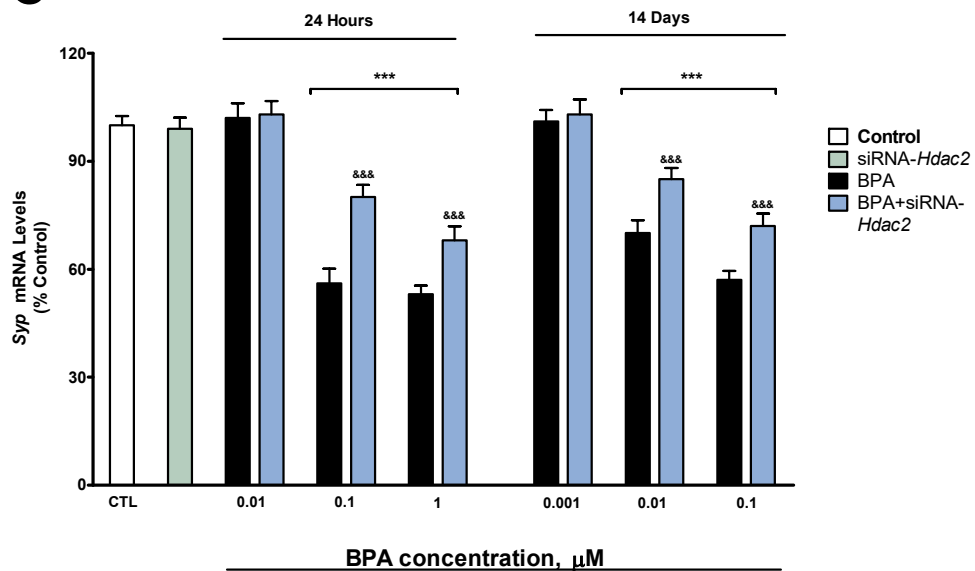**D**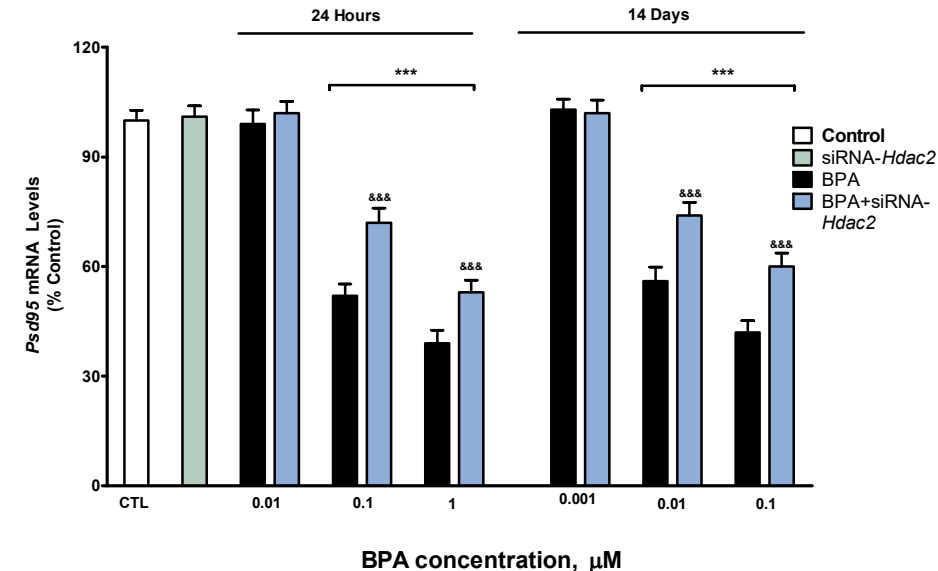

Supplement: Supplementary file 1 [file biology-12-00782-s001.zip › SF4.pdf]
